# Assessment of metabolic interaction between curcumin and tramadol using the isolated perfused rat liver

**DOI:** 10.1016/j.heliyon.2024.e35070

**Published:** 2024-07-23

**Authors:** Maryam Dibaei, Asieh Hosseini, Hoda Lavasani, Banafsheh Kiani-Dehkordi, Mohammadreza Rouini

**Affiliations:** a Razi Drug Research Center, Iran University of Medical Sciences, Tehran, Iran; b Biopharmaceutics and Pharmacokinetic Division, Department of Pharmaceutics, Faculty of Pharmacy, Tehran University of Medical Sciences, Tehran, Iran

**Keywords:** Metabolism, Curcumin, Tramadol, Isolated perfused rat liver

## Abstract

**Introduction:**

The presence of phytochemicals in herbal medicines can lead to herb-drug interactions, altering the levels of these compounds and conventional drugs in the bloodstream by influencing CYP450 activity. Considering curcumin's effect on the CYP enzymes responsible for tramadol metabolism, it is essential to assess the potential interaction between curcumin and tramadol when administered together.

**Materials and methods:**

The pharmacokinetics of tramadol were examined in rats receiving either single or multiple doses of curcumin (80 mg/kg) compared to rats without curcumin treatment. Tramadol liver perfusion was conducted on all rat groups and perfusate samples were collected at specified intervals. Tramadol and its main metabolite were detected using an HPLC system coupled with a fluorescence detector.

**Results:**

Tramadol concentrations were notably higher in the co-administered group compared to both the control and treatment groups. Conversely, lower concentrations of M1 were observed in the co-administered and treatment groups compared to the control group. The AUC_0-60_ parameters for tramadol were as follows: 32944.8 ± 1355.5, 22925.7 ± 1650.1, and 36548.0 ± 2808.4 ng⋅min/ml for the control, treatment, and co-administered groups, respectively. Both the co-administered and treatment groups exhibited a lower AUC_0-60_ of M1 compared to the control group. The lack of significant difference in C_max_ and AUC_0-60_ of M1 between the treatment and co-administered groups suggests that single and multiple doses of curcumin have comparable effects on CYP2D6.

**Conclusions:**

These results indicate a potential for drug interactions when curcumin and tramadol are taken together. Furthermore, the influence of curcumin on tramadol metabolism varied between single and multiple oral administrations of curcumin. Hence, it is vital to highlight this interaction in clinical settings and conduct additional research to fully understand the clinical implications of combining curcumin and tramadol.

## 1 Introduction

**Fig. 1:**
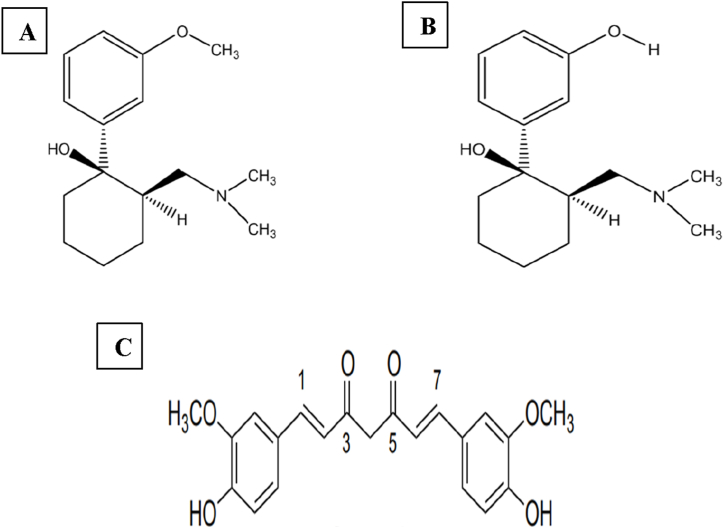
Chemical structure of tramadol (A), O-desmethyltramadol (M1) (B), and curcumin (C).

**Fig. 2:**
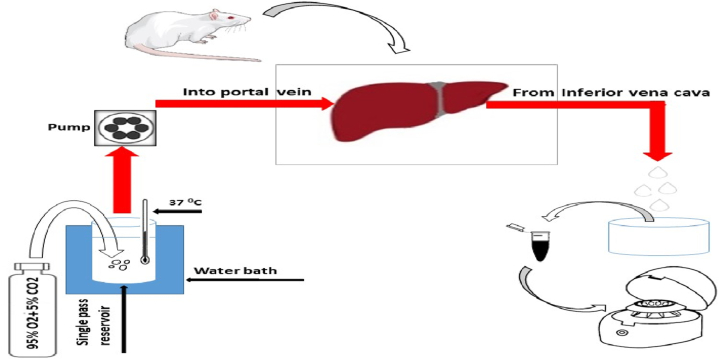
Schematic diagram of isolated perfused rat liver.

**Fig. 3:**
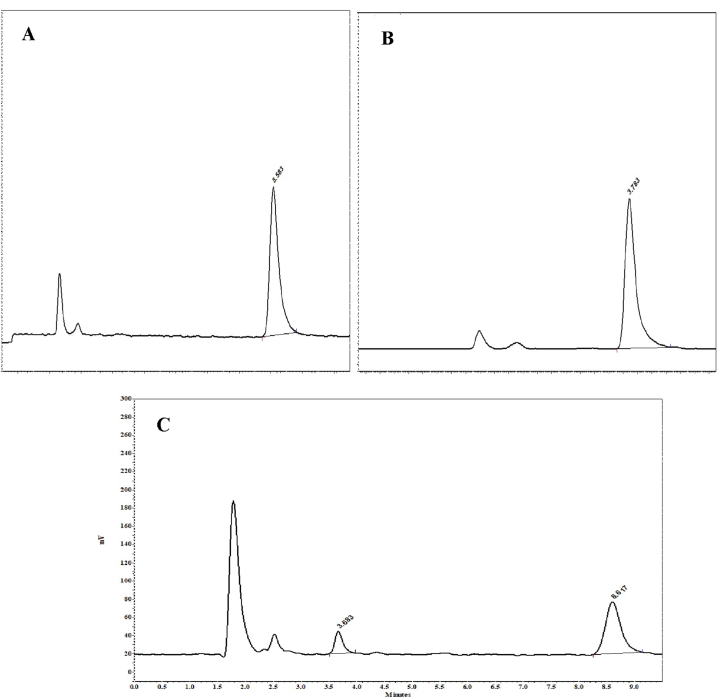
Chromatograms of standard solutions of tramadol (500 ng/ml) (A), M1 (250 ng/ml) (B), and one rat after 5 min of perfusion (C).

**Fig. 4:**
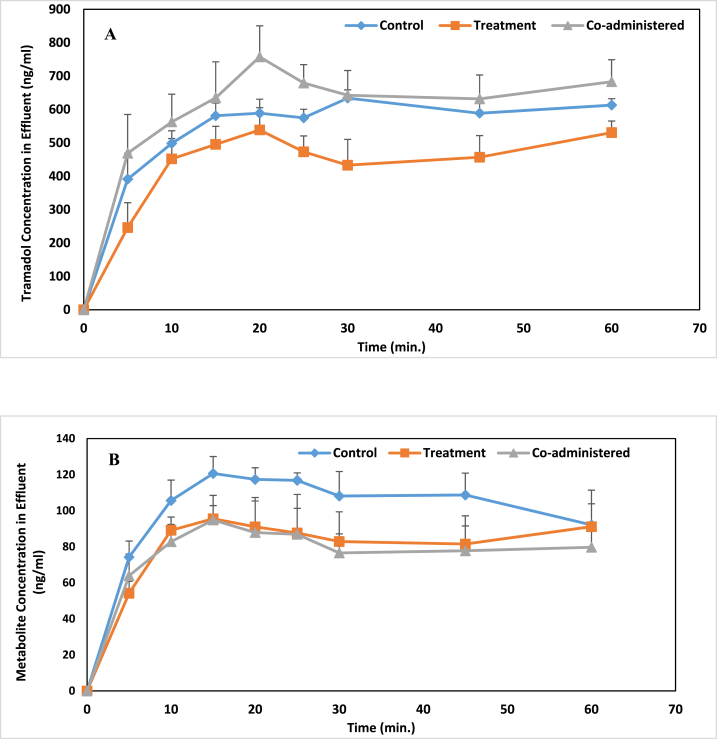
Mean perfusate concentration (Mean ± SEM) vs time profile for tramadol (A) and M1 (B) in liver perfusion experiments. *Control group: Received no curcumin. **Treatment group: Curcumin was administered once daily for seven consecutive days before liver perfusion on the eighth day. ***Co-administered group: Curcumin was administered 1 h prior to tramadol liver perfusion.

**Fig. 5:**
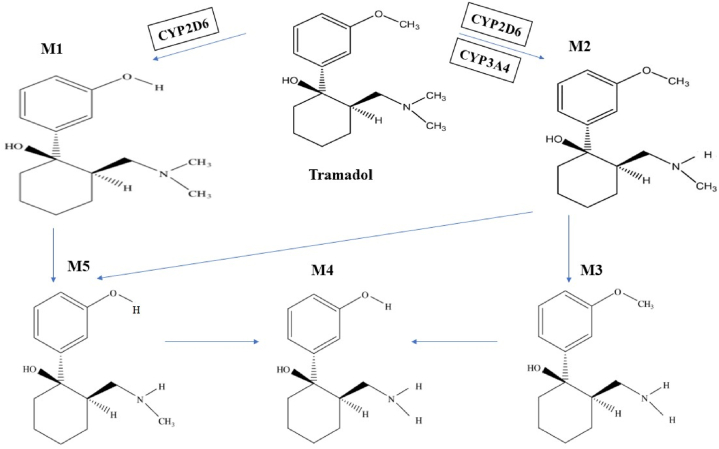
Main metabolites of phase I tramadol metabolism.

Drug metabolism is the process of chemically modifying drug molecules upon their introduction into the body, often resulting in alterations to their therapeutic effectiveness. Cytochrome P450 monooxygenases (CYP) are the primary enzymes responsible for drug metabolism in humans, exhibiting widespread distribution across various tissues and organs, particularly in the liver [1]. These enzymes play a critical role in metabolizing drugs, converting them into either inactive or active metabolites. Additionally, phytochemicals derived from plants serve as substrates for various subtypes within the CYP450 family, potentially influencing the enzymatic activity [2]. Given the multitude of phytochemicals present in herbal medicines, an increased risk of herb-drug interactions exists [3]. Consequently, the simultaneous administration of phytochemicals and conventional drugs can significantly impact the serum levels of both substances by modulating CYP450 activity [2,4]. Notably, among the cytochrome enzymes, CYP3A4 predominates as the most abundant, alongside CYP1A2, CYP2C9, and CYP2D6, collectively contributing to 80 % of the liver's total metabolic activity [5] (see Fig. 1, Fig. 2, Fig. 3, Fig. 4, Fig. 5).

Curcumin (Figure [1(C)]), a hydrophobic polyphenol derived from the rhizome of *Curcuma longa*, constitutes the main curcuminoid in turmeric comprising approximately 77 % curcumin, 17 % demethoxycurcumin, and 3 % bisdemethoxycurcumin [6]. Known for its antibacterial, anti-inflammatory, hypoglycemic, antioxidant, wound-healing, lipid-regulating, antiviral, and anticancer properties, curcumin has minimal hypotoxicity and minimal negative reactions [7,8]. Human studies confirm its safety, tolerability, and lack of significant toxicity [9,10]. Nonetheless, based on *in vitro* and *in vivo* models, curcumin has been associated with various potential drug interactions. Notably, curcumin affects several CYP450 metabolic enzymes, including 1A1, 2B6, 2D6, and 3A4, as well as glutathione-S transferase and p-glycoprotein, indicating its potential to modulate the efficacy of various pharmaceutical agents [2].

In rats, the research explored the interaction between curcumin and amlodipine, revealing a significant increase in the maximum plasma concentration (C_max_), the area under the curve (AUC), and the half-life (t_1/2_) of amlodipine [11]. Conversely, an *in vitro* inhibition study indicated potent inhibition of CYP3A4 and CYP2C8 by curcumin. However, physiologically-based pharmacokinetic (PBPK) simulations suggested minimal to negligible clinical relevance of interactions between curcumin, imatinib, and bosutinib due to the low systemic exposure of curcumin [12]. Pre-administration of nanonized curcumin enhanced the bioavailability of bromocriptine in rats, facilitated through intestinal and hepatic CYP3A enzymes [13]. In another study, simultaneous administration of curcumin, with or without piperine, significantly decreased exposure to tamoxifen and endoxifen. This led to concentrations of endoxifen falling below the efficacy threshold, particularly affecting extensive metabolizer patients, potentially impacting 20–40 % of the patient population [14].

Tramadol (Figure [1(A)]), a synthetic opioid derived from morphine and codeine, has shown effectiveness in managing various types of pain, including incisional and neuropathic pain, as well as acute and chronic pain of moderate to severe intensity [15]. Its mechanism of action involves weak agonism of μ opioid receptors and the inhibition of serotonin and norepinephrine reuptake, thereby reducing pain perception [16,17]. Upon oral administration, tramadol is rapidly and nearly completely absorbed, with the mean peak plasma concentration achieved within 2 h. About 70 % of tramadol is absorbed into the bloodstream, with initial metabolism occurring in the liver [18]. Liver enzymes break down tramadol into O-desmethyltramadol (M1) via CYP2D6 and into N-desmethyltramadol (M2) through CYP 3A4 and 2B6 [19,20]. The O-desmethyltramadol metabolite (M1) (Figure [1(B)]) also acts as a weak activator of μ opioid receptors and is more potent than tramadol [16]. In contrast, N-desmethyltramadol is inactive pharmacologically [19]. These metabolites undergo further transformation into additional forms, which are then conjugated with glucuronic acid. Changes in CYP450 enzyme activity can significantly influence tramadol metabolism. The relationship between tramadol serum levels and its analgesic effects depends on dosage, with serum concentrations of 100–300 ng/ml generally considered effective [21]. Concurrent use with other medications notably reduces tramadol serum levels, ranging from 0.15 to 39 mg/L [22]. Tramadol overdose poses a serious risk of fatalities due to respiratory depression, apnea, cardiac arrest, and seizures [23]. In an *in vitro* study, apatinib significantly inhibited tramadol, with subsequent research showing its ability to enhance the analgesic effects of tramadol and O-desmethyltramadol in rats [19]. Additionally, a drug-drug interaction between tramadol and fluconazole, a moderate CYP3A4 inhibitor, was observed in dogs [24]. Caution is advised when using CYP3A4 inhibitors in patients undergoing tramadol treatment, as they may potentially worsen the serotonergic adverse effects of the drug, possibly leading to psychotic disorders [25].

The isolated perfused liver was utilized to study liver function in the metabolism of xenobiotics. This technique offers several advantages such as maintaining the organ's structure, controlling blood and bile flow, easily manipulating the composition of the perfusion medium, and collecting large number of perfusate samples [26]. Additionally, it allows for conducting experiments without interference from other organs, plasma components, or neural-hormonal impacts [27].

Health practitioners are highly concerned about interactions between herbs and drugs. Curcumin's acceptable safety profile and therapeutic efficacy make it widely used worldwide. When combined with pharmacological agents like tramadol, curcumin can cause pharmacokinetic changes, which may include changes in both C_max_ and AUC due to inhibition of CYP isoenzymes. To comprehensively understand how curcumin affects CYP2D6 and CYP3A4 enzymes and their significance in tramadol metabolism, it is crucial to investigate the potential interaction between curcumin and tramadol when coadministered. Therefore, this study aims to evaluate the pharmacokinetic changes of tramadol following single and repeated oral administration of curcumin using the rat liver perfusion model.

## 2 Materials and methods

### 2.1 Materials

Tehran Chemie company (Tehran, Iran) supplied the pure substances of tramadol and M1. Acetonitrile and methanol were procured from Dr. Mojallali Chemical Co. (Iran). All reagents used were of analytical grade, except for acetonitrile and methanol, which were of HPLC grade.

### 2.2 Animals

Male Wistar rats were obtained from the Laboratory Animals Centre at Tehran University of Medical Sciences. Ethical approvals were obtained from the ethics committee, ensuring compliance with animal research ethics guidelines [28,29]. Rats weighing 180 ± 20 g were used in this study and were kept under standard conditions of temperature (23 ± 2 °C) and humidity (50 ± 10 %), with alternating 12-h light/dark cycles. The study involved three groups of rats, each consisting of five or four individuals, designated as the control, co-administered, and treatment groups.

### 2.3 Experimental design and *in vivo* treatments

Curcumin suspension was prepared immediately before administration using a mixture of olive oil and distilled water. Tramadol liver perfusion was conducted across all three rat groups. The control group received no curcumin and underwent tramadol liver perfusion alone. In the co-administered and treatment groups, rats received an intragastric (i.g.) administration of 80 mg/kg curcumin. In the treatment group, curcumin was administered once daily for seven consecutive days before liver perfusion on the eighth day. In the co-administered group, curcumin was administered 1 h prior to tramadol liver perfusion.

### 2.4 Liver perfusion

The liver perfusion methodology closely followed our previous study [30] (Figure [2]). Initially, rats were anesthetized via intraperitoneal injection of a ketamine–xylazine cocktail (ketamine 100 mg/kg and xylazine 10 mg/kg). Subsequently, cannulation of the portal vein and inferior vena cava was performed. The Krebs-Henseleit buffer, composed of 118 mM NaCl, 4.5 mM KCl, 2.75 mM CaCl2, 1.19 mM KH_2_PO_4_, 1.18 mM MgSO_4_, and 25 mM NaHCO_3_, was oxygenated with a mixture of 95 % O_2_ and 5 % CO_2_ and adjusted to pH 7.4. This buffer was continuously infused at a steady flow rate of 8.3 ml/min via the portal vein using a Terumo® infusion pump. After a 5-min interval from the start of perfusion, the drug-free medium was replaced with a medium containing tramadol (1000 ng/ml), employing a single-mode perfusion technique. The perfusate that passed through the liver was collected from the inferior vena cava. Perfusate samples were collected at 5-min intervals up to 30 min and then at 45 and 60 min.

### 2.5 Equipment and chromatography condition

In this study, an HPLC system consisting of a gradient HPLC pump and a fluorescence detector (Knauer, Berlin, Germany) was utilized, following a previously published method for analysis of tramadol and M1 [31]. Data collected from the detector were processed and analyzed using ChromGate chromatography software (Knauer, Berlin, Germany). Analyte separation was conducted in an isocratic mode, employing a Chromolith™ C18 column (100 mm × 4.6 mm i.d.) packed with 5 μm particles. The mobile phase consisted of a mixture of methanol and water (19:81, v/v), adjusted to pH 2.5 using phosphoric acid and filtered through a 0.45 μm filter before use. The flow rate was maintained at 2 mL/min at ambient temperature. Tramadol detection was performed using a fluorescence detector, with excitation and emission wavelengths of 275 and 302 nm, respectively.

### 2.6 Kinetic parameters and statistical analysis

The areas under the concentration-time curves (AUC_0-60_) for tramadol and M1 were calculated using the trapezoidal rule. Subsequently, metabolic ratios were computed by dividing the AUC_0-60_ of each metabolite by that of tramadol. Statistical analysis of the data performed using a utilizing one-way analysis of variance (ANOVA) and Holm-Sidak method with SigmaPlot software. A *p-value* of ≤0.05 was considered statistically significant. The results were expressed as mean ± standard error of the mean (SEM).

## 3 Results

The approved assay conditions were verified following FDA guidelines to ensure specificity, linearity, accuracy, and precision [32]. Both analytes demonstrated proper linearity with good correlation coefficients, and met the acceptable criteria of <15 % for accuracy and precision on both intra- and inter-day measurements. Figure [3(A-C)] Showed chromatograms of standard solutions of tramadol, M1, and one rat after 5 min of perfusion. Using the calibration curves of tramadol and its metabolite, we determined their concentrations at various time points post-liver perfusion across the studied groups. Figure [4 (A-B)] illustrates the temporal profiles of tramadol and M1 in the effluent for the different experimental groups in the liver perfusion experiments. As shown in the figure, tramadol concentrations in the effluent follow the trend: co-administered < control < treatment groups. Conversely, the control group exhibited the highest levels of M1 concentration compared to the co-administered and treatment groups. Table 1 presents the pharmacokinetic parameters for tramadol and M1 across the different experimental groups. The C_max_ values for tramadol were 659.1 ± 34.2, 620.2 ± 17.2, and 836.6 ± 30.2 ng/ml for the control, treatment, and co-administered groups, respectively. Additionally, the C_max_ values for the metabolite are 131.5 ± 6.1, 121.3 ± 4.0, and 118.6 ± 15.6 ng/ml for the control, treatment, and co-administered groups, respectively. The metabolic ratios of M1 were measured as 0.19 ± 0.02 for the control group, 0.18 ± 0.02 for the treatment group, and 0.16 ± 0.03 for the co-administered group.

**Table 1:** The pharmacokinetic parameters (Mean ± SEM) for tramadol and M1 in different groups.

Group	Tramadol		M1		
	C_max_ (ng/ml)	AUC_0-60_ (ng⋅min/ml)	C_max_ (ng/ml)	AUC_0-60_ (ng⋅min/ml)	Metabolic Ratio
Control^a^	659.1 ± 34.2	32944.8 ± 1355.5	131.5 ± 6.1	6075.9 ± 431.2	0.19 ± 0.02
Treatment^b^	620.2 ± 17.2	22925.7 ± 1650.1	121.3 ± 4.0	4262.3 ± 612.1	0.18 ± 0.02
Co-administered^c^	836.6 ± 30.2	36548.0 ± 2808.4	118.6 ± 15.6	4610.9 ± 615.8	0.16 ± 0.03

a Control group: Received no curcumin.

b Treatment group: Curcumin was administered once daily for seven consecutive days before liver perfusion on the eighth day.

c Co-administered group: Curcumin was administered 1 h prior to tramadol liver perfusion.

All pairwise multiple comparison procedures with an overall significance level of 0.05 indicated no difference in the C_max_ of M1 among three groups. However, the C_max_ of tramadol in the co-administered group exhibited a significant difference compared to the treatment (p = 0.002) and control (p = 0.006) groups, which did not show statistical disparity (p = 0.393). The AUC_0-60_ parameters for tramadol were 32944.8 ± 1355.5, 22925.7 ± 1650.1, and 36548.0 ± 2808.4 ng⋅min/ml for the control, treatment, and co-administered groups, respectively. For the metabolite, the corresponding AUC_0-60_ parameters were 6075.9 ± 431.2, 4262.3 ± 612.1, and 4610.9 ± 615.8 ng⋅min/ml for the control, treatment, and co-administered groups, respectively. The results revealed a significant difference in the AUC_0-60_ parameters of tramadol among treatment and control groups (p = 0.005) and treatment and co-administered groups (p = 0.001) with no significant difference between co-administered and control groups (p = 0.218). Furthermore, a notable distinction was not observed in the AUC_0-60_ of the metabolite between the treatment and co-administered groups (p = 0.352). However, significant differences noted between the control and co-administered groups (p = 0.042), as well as between the control and treatment groups (p = 0.021). There was no significant difference in the metabolic ratios among three groups. This lack of difference could be attributed to the effects of curcumin on various enzymes involved in tramadol metabolism, as well as the different inhibitory and inductive effects of curcumin on these enzymes with single and multiple doses.

## 4 Discussion

Tramadol undergoes hepatic metabolism and is primarily eliminated through the kidneys, making these organs susceptible to potential tramadol toxicity [33]. After oral administration of tramadol, it was rapidly and completely metabolized. O-demethylation, N-demethylation, cyclohexyl oxidation, oxidative N-dealkylation, dehydration, and conjugation were used to form the metabolites. Pathways 1–3 are significant steps that produce seven O-desmethyl/N-desmethyl and hydroxy-cyclohexyl metabolites in significant amounts. O-desmethyl tramadol (M1), N-desmethyl tramadol (M2), N,N-didesmethyl (M3), N,N,O-tridesmethyl tramadol (M4) and N,O-didesmethyl tramadol (M5), and uncharacterized conjugates were reported (figure [5]). *In vitro* metabolism of tramadol was performed and CYP2D6 and CYP3A4 were identified as the major cytochrome P450 isoforms [34]. CYP2D6 is responsible for catalyzing the O-demethylation of tramadol to M1, which is the primary analgesic metabolite. Similarly, CYP2B6 and CYP3A4 are involved in the N-demethylation of tramadol to M2 [35,36]. The Metabolite M1 is more potent than tramadol in binding to μ-opioid receptors and in providing pain relief [37].

Studies have noted that hepatotoxicity and nephrotoxicity can occur with either a single effective analgesic dose of tramadol or when taken at the maximum recommended daily dosage [38]. Moreover, in a separate study, tramadol administration showed dose-dependent toxic effects on the brain, kidneys, and liver in rats [39]. Previous research has suggested that the administration of curcumin may alleviate the harmful effects of tramadol on the glomerulus, proximal tubule, and distal tubule of the kidneys [40]. Another study found that turmeric extract reduced the toxicity caused by tramadol in mice [41]. Furthermore, research showed that giving curcumin to rats before exposing them to tramadol provided protective effects on their overall health and kidney function against tramadol toxicity [42].

CYP450 enzymes are crucial in activating and deactivating various external and internal substances [43,44]. Curcumin has been found to competitively inhibit CYP3A4 and CYP2B6 while showing noncompetitive inhibition against CYP2D6 [45]. Therefore, considering the involvement of these enzymes in tramadol metabolism, it is crucial to consider the potential impact of curcumin on CYP2D6, CYP2B6, and CYP3A4 enzymes. Consequently, evaluating the interaction between curcumin and tramadol when administered together is essential. This assessment should anticipate alterations in tramadol concentration, enabling appropriate adjustments in tramadol dosage. The objective of this study was to evaluate the pharmacokinetic changes of tramadol following single and repeated oral doses of curcumin using a rat liver perfusion model. Such investigations can provide valuable insights for the clinical co-administration of curcumin and tramadol.

According to our findings, the lowest and highest concentrations of tramadol observed in the effluent during liver perfusion experiments were associated with the treatment and co-administered groups, respectively. Initially, it was expected that inhibiting tramadol-metabolizing enzymes in the liver via curcumin result in the lowest tramadol levels in the control group, which did not receive curcumin. However, contrary to expectations, the order of magnitudes of C_max_ and AUC_0-60_ for tramadol was highest in the co-administered group, followed by the control group, and finally, the treatment group. Although higher concentrations were for tramadol observed in the co-administered group compared to the control group, the difference in AUC_0-60_ for tramadol between these two groups was not statistically significant. However, multiple administrations of curcumin showed significant effects on tramadol concentrations compared to the other two groups. Notably, curcumin administration not only altered tramadol metabolism but also led to differences in tramadol metabolism between single and repeated administrations of curcumin groups. It appears that the relative inhibition of CYP3A4, CYP2B6, and CYP2D6 by curcumin during single administration resulted in decreased tramadol metabolism, leading to the highest C_max_ and AUC_0-60_ of tramadol observed in the co-administered group. However, with repeated curcumin administration, alongside its inhibitory effect, metabolism was also induced. This induction was evidenced by increased CYP3A4 expression due to seven consecutive days of curcumin administration in the treatment group. Available data suggest that curcumin may influence CYP3A transcription via the pregnane X receptor (PXR) [46]. The higher levels of M1 concentration and AUC_0-60_ observed in the control group were attributed to the curcumin's inhibition of CYP2D6 in the other two groups. The absence of a significant difference between C_max_ and AUC_0-60_ of M1 in the treatment and co-administered groups indicates that both single and multiple administrations of curcumin have equivalent effects on CYP2D6.

Numerous *in vivo* and *in vitro* animal studies have highlighted the significant impact of curcumin on the activity of various drug-metabolizing enzymes through mechanisms like down-regulation, induction, or direct inhibition. For example, Hsieh et al. noted that oral intake of curcumin reduced the absorption of everolimus, a probe substance for P-gp/CYP3A4, mainly due to the marked enhancement of CYP3A4 activity by curcumin metabolites [47]. *In vivo* experiments showed that rats given repeated curcumin administration exhibited a notable increase in hepatic and renal CYP3A levels, while intestinal CYP3A enzymes were down-regulated [39]. Additionally, an *in vitro* study on CYP inhibition found that curcumin inhibited testosterone metabolism by CYP3A. However, the effect of a single oral dose of curcumin on the pharmacokinetics of buspirone in rats was minimal [48]. In contrast, according to Price et al., curcumin did not significantly affect the mRNA expression of CYP2B6 and CYP3A in *in vitro* experiments [49]. Similarly, other reports have indicated that curcumin had no significant effect on mRNA expression in various cells, including human hepatocytes and HepG2 cells [50,51]. Nonetheless, our research suggests that both single and multiple doses of curcumin could alter the metabolism of tramadol.

Despite curcumin's notable inhibitory effects on M1, the main metabolite responsible for pharmacological effects, and the resulting decrease in tramadol concentration with multiple administrations, the observed reduction in tramadol toxicity may be due to lower tramadol exposure rather than the protective effects of curcumin. Moreover, decreased tramadol exposure could have adverse implications for its effectiveness in pain management. Further evaluation is needed to assess curcumin's potential in mitigating hepatotoxicity and renal toxicity induced by tramadol. Additional research is necessary to understand the exact mechanisms behind these findings and determine the clinical implications for pain management strategies involving tramadol and curcumin co-administration.

## 5 Conclusion

In this study, using a rat liver perfusion model, we examined how tramadol's pharmacokinetics changed after single or repeated oral doses of curcumin. We found distinct changes in tramadol concentrations between the single and multiple curcumin administrations. Notably, multiple curcumin doses significantly reduced tramadol concentration compared to the other groups. Furthermore, both the treatment and co-administered groups showed decreased levels of M1 concentration and AUC_0-60_ due to curcumin's inhibition of CYP2D6, indicating similar effects of single and multiple curcumin doses on CYP2D6 activity. In conclusion, understanding the interaction between curcumin and tramadol in clinical settings is essential, and further research is needed to clarify its implications for human health.

## Ethics statement

Ethical and legal approvals were obtained from the ethics committee of Iran University of Medical Sciences (Approval Code: IR.IUMS.AEC.1401.006).

## Funding

This study was supported by Iran university of medical sciences.

## Data availability statement

Data associated with the study has not been deposited into a publicly available repository. Data supporting the findings of this study are available from the corresponding authors upon reasonable request.

## CRediT authorship contribution statement

**Maryam Dibaei:** Writing – original draft, Validation, Methodology, Investigation, Formal analysis, Conceptualization. **Asieh Hosseini:** Writing – review & editing, Supervision, Methodology. **Hoda Lavasani:** Writing – review & editing, Methodology, Investigation. **Banafsheh Kiani-Dehkordi:** Writing – review & editing, Investigation. **Mohammadreza Rouini:** Writing – review & editing, Supervision, Project administration, Methodology, Data curation, Conceptualization.

## Declaration of competing interest

The authors declare that they have no known competing financial interests or personal relationships that could have appeared to influence the work reported in this paper.
